# Profil épidémiologique et pronostic de l’insuffisance cardiaque aiguë: expérience du service d’accueil des urgences de l’hôpital Charles Nicole de Tunis de 2013 à 2014

**DOI:** 10.11604/pamj.2019.33.251.17207

**Published:** 2019-07-24

**Authors:** Sarra Jouini, Héla Manai, Olfa Slimani, Hana Hedhli, Fatma Hebaieb, Mohamed Mezghanni, Asma Aloui, Rym Ben Kaddour

**Affiliations:** 1 Hôpital Charles Nicolle, Service des Urgences, Tunis, Tunisie; 2 Université Tunis El Manar, Faculté de Médecine de Tunis, Tunis, Tunisie; 3 Hôpital Charles Nicolle, Service de Gynécologie Obstétrique,Tunis, Tunisie

**Keywords:** Insuffisance cardiaque aiguë, urgences, traitement, mortalité, Acute high failure, emergency department, treatment, mortality

## Abstract

**Introduction:**

l'Insuffisance Cardiaque Aiguë (ICA) correspond à une entité syndromique spécifique, regroupant plusieurs tableaux cliniques hétérogènes; fréquemment rencontrées aux urgences. L'objectif de cette étude a été de décrire les caractéristiques épidémiologiques, cliniques, thérapeutiques et pronostiques des patients admis aux urgences pour ICA.

**Méthodes:**

Nous avons mené une étude prospective descriptive dans un service d'accueil des urgences qui a inclus tous les patients admis pour ICA. Nous avons étudié les caractéristiques épidémiologiques, cliniques, thérapeutiques et pronostiques chez ces patients.

**Résultats:**

Nous avons inclus 180 patients pour ICA ayant entrainé une hospitalisation dans le service d'urgence. Le sexe ratio a été de 1,27. L'âge moyen a été de 66±12 ans. Quatre vingt deux pour cent des patients étaient hypertendus et 69% étaient diabétiques connus. Les étiologies de décompensation étaient essentiellement une poussée hypertensive chez 61,7% des patients, un syndrome coronaire aigu chez 24%. Le support respiratoire a été assuré essentiellement par la CPAP (*Continuous Positive Airway Pressure*) dans 73,3% des cas. Le traitement pharmacologique a été à base de dérivés nitrés dans 70% et de diurétique dans 40,5% des cas. Le taux de récidive de l'insuffisance cardiaque aiguë à un mois a été de 21,7% (n=39 patients), et celui de la mortalité à 3 mois a été de 13,3%.

**Conclusion:**

l'ICA vue au niveau des urgences est essentiellement sous forme hypertensive. Le traitement est basé essentiellement sur la CPAP, les vasodilatateurs et les diurétiques. Le taux de récidive était important, et la mortalité était aussi élevée.

## Abstract (en)

**Introduction:**

Acute Heart Failure (AHF) is a specific syndromic disorder grouping several heterogeneous clinical conditions frequently seen in the emergency department. This study aimed to describe the epidemiological, clinical, therapeutic and prognostic features of patients with AHF admitted to the emergency department.

**Methods:**

We conducted a prospective, descriptive study in the emergency department. It included all patients admitted with AHF. We studied the epidemiological, clinical, therapeutic and prognostic features of these patients.

**Results:**

The study enrolled 180 patients with AHF admitted to the emergency department. Sex ratio was 1.27. The average age of patients was 66±12 years. Eighty-two percent of patients were hypertensive and 69% were known diabetic patients. The causes of decompensation included primarily hypertensive crisis (61.7% of patients), acute coronary syndrome (24% of patients). Respiratory support was mainly provided by CPAP (Continuous Positive Airway Pressure) in 73.3% of cases. Pharmacological treatment was based on nitrate derivatives (70% of cases) and diuretic (40.5% of cases). Acute heart failure incidence at one month was 21.7% (n=39 patients) and mortality rate at 3 months was 13.3%.

**Conclusion:**

Patients with AHF treated in the emergency department mainly had hypertensive crisis. Treatment is primarily based on CPAP, vasodilators and diuretics. Recurrence rate and mortality rate were high.

## Introduction

L'insuffisance cardiaque aiguë (ICA), correspond à une entité syndromique spécifique, regroupant plusieurs tableaux cliniques hétérogènes aussi bien sur le plan physiopathologique, évolutif, pronostique et thérapeutique [1, 2]. Les syndromes d'insuffisance cardiaque aiguë (SICA) constituent un problème majeur de santé publique, c'est une pathologie qui a été responsable de plus de 26 millions d'hospitalisations dans le monde et d'un cout annuel élève [3-5]. D'un autre côté, 80% des patients hospitalisés pour insuffisance cardiaque aiguë le sont à partir des urgences [6]. Le pronostic de l'insuffisance cardiaque aiguë reste lourd avec un taux de récidive entre 24% à un mois et 46% à deux mois et un taux de mortalité qui varie entre 7% et 11% à trois mois [2, 7, 8]. Les sociétés savantes se sont basées sur les données récentes d'études multicentriques internationales observationnelles, pour proposer des classifications cliniques et émettre des guidelines de prise en charge clinique et thérapeutique afin d'améliorer la prise en charge des SICA durant les phases pré hospitalières et hospitalières précoces [9-12]. Les services d'urgence jouent un rôle primordial dans l'amélioration du pronostic des syndromes d'insuffisance cardiaque aiguë à travers une prise en charge précoce et standardisée conformément aux recommandations [9-12]. L'objectif de cette étude a été de décrire les caractéristiques épidémiologiques, cliniques, thérapeutiques et pronostiques des patients admis aux urgences pour insuffisance cardiaque aiguë.

## Méthodes

Il s'agissait d'une étude prospective, observationnelle et descriptive qui s'est déroulée aux urgences de l'hôpital Charles Nicolle de Tunis sur une période s'étendant sur 18 mois (mars 2013-septembre 2014). Nous avons inclus les patients qui s'étaient successivement présentés aux urgences pour dyspnée compatible avec un tableau d'insuffisance cardiaque aiguë. Non pas été inclus les patients âgés de moins de 18 ans et les femmes enceintes. Le diagnostic d'ICA a été porté conformément aux critères de l'*Europeen Society of Cardiology* sur un faisceau d'arguments anamnestiques, cliniques, électro-cardiographiques et radiologiques [9]. Selon la gravité de la présentation clinique initiale, les patients étaient pris en charge soit en salle d'Accueil des Urgences Vitales (SAUV) en cas de détresse respiratoire extrême puis en Unité de Surveillance Rapprochée (USR) soit en Unité d'Hospitalisation de Courte Durée (UHCD) en l'absence de critères de gravité. Le protocole thérapeutique a été standardisé et a reposé sur plusieurs volets.

**Le support respiratoire comportait plusieurs modalités:** CPAP (*Continuous Positive Airway Pressure*); BIPAP (*Bilevel Positive Airway Pressure*) et oxygénothérapie au masque (masque facial simple ou masque à oxygène haute concentration). Les vasodilatateurs avec la prescription du Dinitrate d'Isosorbide (Risordan^®^) qui a été faite par voie intraveineuse, au pousse seringue électrique à des posologies adaptées aux chiffres de la pression artérielle systolique.

**Les diurétiques:** un premier bolus de Furosémide (lasilix^®^) a été donné à la dose de 40mg en intraveineux direct chez les patients en insuffisance cardiaque aiguë avec des signes de congestion systémique. Les doses suivantes ont été ajustées en fonction de la clairance de la créatinine, des doses antérieures de diurétiques reçus par le malade, de l'importance des signes de congestion systémique et de l'évolution clinique du patient. On a noté la mortalité intrahospitalière, à un mois et à trois mois, en plus de la récidive de l'ICA dans le mois.

**Analyse statistique:** l'acquisition des données et l'étude statistique ont été réalisées au moyen du logiciel SPSS 19.0 for Windows. Nous avons mené une étude descriptive avec calcul des fréquences simples et des fréquences relatives pour les variables qualitatives. Calcul des moyennes, des médianes, des écarts-types, et de l'étendue pour les variables quantitatives.

**Aspects éthiques:** nous avons mené une étude observationnelle descriptive; aucune intervention à visée exploratrice ou thérapeutique n'a été imposée. Le protocole thérapeutique exposé a été celui du service. Les patients et leurs familles étaient d'accord pour afficher leurs numéros de téléphone dans le dossier afin d'assurer le suivi.

## Résultats

**Figure 1:**
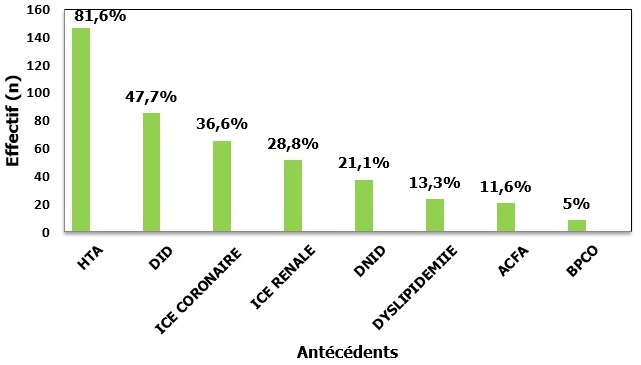
Répartition des patients selon les antécédents (ACFA: arythmie complète par fibrillation auriculaire; BPCO: broncho-pnuemopathie chronique obstructive; DID: diabète insulinodépendant; DNID: diabète non insulinodépendant; HTA: hypertension artérielle; ICE coronaire: insuffisance coronaire; ICE rénal: insuffisance rénale)

**Figure 2:**
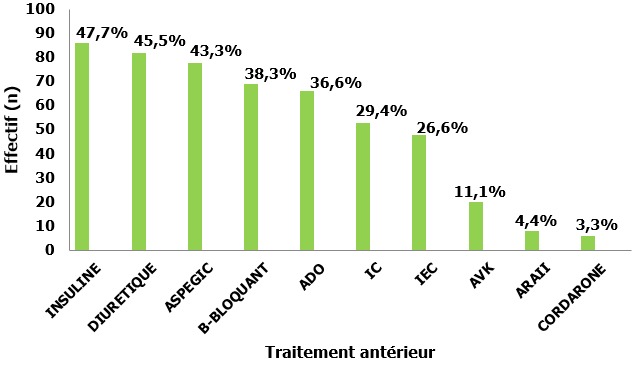
Répartition des patients selon le traitement antérieur (AVK: anti vitamine k; ARAII: antagonistes des l'angiotensine II; ADO: anti diabètiques oraux; IEC: inhibiteurs de l'enzyme de conversion; IC: inhibiteurs calciques)

Durant la période de l'étude (mars 2013-septembre 2014), 180 patients ont été inclus pour insuffisance cardiaque aiguë ayant entrainé une hospitalisation dans le service d'urgence. Cent-un patients étaient de sexe masculin (56,1%) et 79 étaient de sexe féminin (43,9%), avec une sex-ratio= 1,27. La moyenne d'âge a été de 66,7±12 ans avec des extrêmes allant de 20 à 95 ans. Cent-trente patients (72,2%) étaient âgés de plus de 60 ans. Neuf patients (5%) seulement étaient sans antécédents médicaux pathologiques connus; 171 patients (95%) avaient au moins un antécédent médical. Les antécédents les plus retrouvés étaient l'hypertension artérielle et le diabète. Les Figure 1 et Figure 2 schématisent les principaux antécédents et médicaments pris par les patients, (Figure 1, Figure 2). Le tableau clinique initial était dominé par le tableau d'insuffisance cardiaque aiguë hypertensive qui représentait 85% des cas. Les données cliniques sont résumées sur le Tableau 1.

**Tableau 1:** Paramètres cliniques et gazométriques à l’admission

	Moyenne +/-ET	Valeurs extrêmes
PAS (mmHg)	184 +/- 42	100 - 300
PAD (mmHg)	98 +/- 21	40 - 180
FR (cycles/min)	32 +/- 7	10 - 50
FC (battements/min)	109 +/- 27	20 - 220
SpO2 (%) en air ambiant	77,5 +/- 13	23 - 92
EVA dyspnée (cm)	7,38 +/- 2	2 - 10
pH	7,27 +/- 0,18	6,52 - 7,54
PaO2 (mmHg)	70,9 +/- 30	24 - 257*
Lactate (mmol/L)	2,59 +/- 2,34	0,3 - 11,2

EVA: Echelle Visuelle Analogique; FR: fréquence respiratoire; FR: fréquence cardiaque; PAS: Pression artérielle systolique; PAD: pression artérielle diastolique; PaO2: Pression artérielle en oxygène; SpO2: saturation pulsée en oxygène.

* : Certaines gazométries ont été prélevées sous FiO2 > 21%.

Des signes de congestion pulmonaire et systémique étaient présents avec des râles crépitants chez 173 patients (96,1%) et des râles sibilants chez 16 patients (8,9%); les œdèmes des membres inférieurs chez 66 patients (36,7%), une turgescence des jugulaires et un reflux hépato jugulaire chez 5 patients (2,8%). Dix-sept patients (10,5%) avaient des signes d'hypoperfusion périphérique: extrémités froides et marbrures. Les causes de décompensations retenues étaient une poussée hypertensive chez 61,7% des patients, un syndrome coronaire aigu chez 24% des patients, une cause infectieuse chez 15,6% des patients et un trouble du rythme chez quatre patients. Concernant le support respiratoire, quatre patients (2,2%) avaient nécessité le recours à l'intubation et la mise sous ventilation mécanique. La modalité majeure d'oxygénothérapie était représenté par la CPAP (Continuons Positive Airway Pressure); cent trente-deux (73,7%) patients ont été mis sous CPAP avec une durée moyenne de 4,55±2,39 heures et des extrêmes allant de 0 à 10 heures. Le traitement pharmacologique était à base de dérivée nitrée utilisée chez 70% des patients avec une dose moyenne de 27±30mg (médiane à 24mg et les extrêmes 6 et 310mg); et soixante treize patients (40,5%) avaient reçu un traitement par diurétique en intraveineux avec une dose moyenne de 106±82 (les extrêmes 0 à 500mg). Les patients ont été hospitalisés initialement dans les différents secteurs du service des urgences: l'unité de surveillance rapprochée (USR) pour les patients les plus graves et l'unité d'hospitalisation de courte durée (UHCD) pour les patients stables. Cent trente-neuf patients (77,22%) ont été mis sortant directement à partir des urgences. La durée moyenne d'hospitalisation aux urgences a été de 16±2 heures avec des extrêmes de 3 à 120 heures. Le taux de récidive de l'insuffisance cardiaque aiguë à un mois a été de 21,7% (n=39 patients). Le Tableau 2 représente les taux de mortalité intra hospitalière, à un mois et à trois mois (Tableau 2).

**Tableau 2:** Récidive et mortalité de l’insuffisance cardiaque aiguë

	Nombre (%)
Récidive à un mois	39 (21,6)
Mortalité intra hospitalière	6 (3,3)
Mortalité à un mois	21 (11,6)
Mortalité à trois mois	24 (13,3)

## Discussion

Cent quatre vingt patients hospitalisés pour insuffisance cardiaque aiguë ont été inclus, les antécédents principaux étaient l'hypertension artérielle et le diabète. Le traitement était basé essentiellement sur la CPAP, les vasodilatateurs et les diurétiques. Le taux de récidive était important, et la mortalité était aussi élevée. Cette étude présente beaucoup de points forts notamment l'aspect prospectif, en plus notre étude a été faite dans un service d'urgence médico-chirurgicale polyvalent qui représente actuellement le lieu de prise en charge et d'hospitalisation des patients en ICA. Des points faibles sont à noter dans cette étude notamment le nombre de patients inclus: bien que le déroulemenent monocentrique de l'étude a fait que la population étudiée soit une population homogène mais le nombre limité des patients inclus reste un point faible dans cette étude. L'absence d'évaluation écho cardiographique de la fonction ventriculaire gauche et l'absence de dosage du BNP et NT Pro BNP, outil diagnostique dans l'insuffisance cardiaque aiguë mais aussi qui ont une valeur pronostique.

En comparant les données démographiques et épidémiologiques de notre étude avec les résultats de la littérature, on remarque que les sujets inclus dans cette étude étaient plus jeunes avec une moyenne d'âge de 66 ans pour 70 à 74 ans dans la littérature [8, 12, 13]. Une légère prédominance masculine avec sex-ratio de 1,27. L'antécédent de diabète était présent chez 69% des patients dans cette série par rapport à 30% à 40% dans la littérature [8, 12, 13]. La différence d'âge constatée peut être expliquée par les faits suivants: les pays en voie de développement ont une population plus jeune par rapport aux pays développés et en plus le lieu de recrutement des patients au niveau du service des urgences et non pas dans des services hospitaliers comme c'est le cas pour les études sus-citées. Le taux élevé des diabétiques constaté dans notre étude peut être en rapport avec l'augmentation de l'incidence du diabète dans la population générale: transition épidémiologique avec modification du mode de vie (obésité, sédentarité) et l'augmentation du nombre de patients sous médicaments connus pour avoir un effet diabétogène comme les diurétiques, les bétabloquants et les corticoïdes [14].

En comparaison avec les données rapportées dans la littérature, les patients inclus dans notre étude se caractérisaient par des chiffres plus élevés de pression artérielle et des signes de congestion pulmonaire plus fréquents compatibles avec une ICA hypertensive. Le tableau d'ICA hypertensive était présent dans 85% des cas dans cette série contre 50% dans la littérature [8, 12, 13]. La prévalence élevée du tableau clinique d'ICA hypertensive dans notre étude s'explique par la gravité de cette forme clinique où prédominent les signes de congestion pulmonaire résultant en une insuffisance respiratoire aiguë imposant le recours immédiat aux services d'urgence. Les patients hospitalisés dans les services de cardiologie présentent des tableaux moins graves correspondant à des décompensations d'insuffisance cardiaque chronique. Les recommandations internationales optent pour une prise en charge thérapeutique rapide voire même dès la phase pré hospitalière [15]. La ventilation spontanée (VS) en pression positive, appelée aussi CPAP (*Continuous Positive Airway Pressure*), et l'aide inspiratoire (AI) associée à une PEP (VS-AI-PEP) appelée aussi BIPAP (*Bilevel Positive Airway Pressure*), font partie de l'arsenal thérapeutique de la prise en charge de l'ICA aux urgences [16, 17]. La CPAP est le mode ventilatoire le plus utilisé aux urgences dans les ICA.

Dans notre étude, nous avons utilisé le dispositif de CPAP de BOUSSIGNAC^®^. Le taux de recours à l'intubation et la mise sous ventilation mécanique dans notre étude était inférieur à celui décrit dans la littérature (2,2% contre 5% à 16% dans la littérature) [8, 13, 18]. Ceci peut être expliqué par la précocité du traitement; dans notre étude, le traitement a été instauré dès l'admission du patient au service des urgences. Le support ventilatoire a été adapté selon la gravité du tableau clinique conformément au schéma thérapeutique proposé dans les recommandations [15]. Le bénéfice clinique de l'administration de diurétique en cas d'ICA est universellement accepté, les diurétiques de l'anse constituent le premier choix en cas d'ICA [19, 20]. Par leur effet sur l'excrétion rénale d'eau et de NaCl et un effet veinodilatateur immédiat, ils diminuent efficacement la précharge. L'administration initiale d'une dose de 20mg à 40mg en intraveineux est recommandée pour sa rapidité d'action (5-30 min) [15]. La fréquence d'utilisation des diurétiques dans notre étude ne rejoint pas celle rapportée dans la littérature où les diurétiques étaient les premiers agents prescrits dans les ICA (87% à 90% dans la littérature contre 40,6% dans notre étude) [8, 13, 18]. La forme clinique la plus fréquente admise aux urgences était l'insuffisance cardiaque aiguë hypertensive; ces patients sont en général normo voire même hypovolémique, même si le tableau clinique reflète une augmentation des pressions de remplissage ventriculaire gauche. L'adjonction des diurétiques sur ce terrain aggrave donc l'hypovolémie, précipite l'activation neuro-hormonale du Système Rénine-Angiotensine-Aldostérone (SRAA) et du système nerveux sympathique [21]. Les diurétiques constituent une thérapeutique de premier choix dans l'insuffisance cardiaque aiguë normotensive en présence de signes de surcharge systémique et pulmonaire.

Les dérivés nitrés sont indiqués précocement dès la phase pré hospitalière, la voie sublinguale est utile dans ce contexte [15]. Les dérivés nitrés restent les plus utilisés, ils sont indiqués dans l'ICA hypertensive, normotensive ou associée à un syndrome coronaire aigu; les doses sont modulées afin de préserver une pression artérielle systolique PAS ≥110 mmHg. En effet, ils permettent de diminuer les résistances vasculaires systémiques et les pressions de remplissage du ventricule gauche et droit [22]. Le recours plus fréquent aux dérivés nitrés dans notre étude s'explique par la fréquence élevée des formes d'ICA hypertensive. Dans la littérature, le taux de mortalité intra hospitalière varie entre 4% et 7% [2, 8, 13, 23, 24]. La mortalité intra-hospitalière immédiate a été chiffrée à 11% dans le registre ALARM-HF [18]. Cette mortalité très élevé était expliquée par la fréquence des patients admis en état de choc cardiogénique (12% versus moins de 4% dans les autres registres).

La prise en charge précoce des patients en ICA notamment au niveau des services des urgences était associée à un taux de mortalité intra hospitalière plus bas [25]. Dans notre série la mortalité intrahospitalière a été de 3,3%. La mortalité à trois mois varie entre 7% et 11% dans l'ICA [2, 8, 14]. Une des particularités des SICA est le taux élevé de récidives responsable d'une fréquence élevée de réhospitalisation. Le taux enregistré dans cette série a été de 21,7% rejoignant celui de la littérature de 22% à 30% [2, 8, 12]. Les syndromes d'insuffisance cardiaque aiguë restent toujours du domaine de la recherche médicale notamment concernant les facteurs pronostiques vue l'importante morbi-mortalité de cette pathologie; en plus du traitement pharmacologique avec les nouveaux vasodilatateurs.

## Conclusion

L'incidence de l'insuffisance cardiaque aiguë ne cesse d'augmenter parallèlement au vieillissement de la population et à l'amélioration de la prise en charge des maladies cardiovasculaires dont le stade ultime est l'insuffisance cardiaque. Le tableau ICA hypertensive est fréquemment observé au niveau des services des urgences à cause de la défaillance respiratoire associée. Le traitement est basé essentiellement sur la CPAP, les vasodilatateurs et les diurétiques. Une prise en charge adéquate et adaptée initiée précocement au niveau des urgences permet de diminuer la morbi-mortalité de cette pathologie.

### Etat des connaissances actuelles sur le sujet

- L'incidence de l'insuffisance cardiaque aiguë ne cesse d'augmenter parallèlement au vieillissement de la population et l'amélioration de la prise en charge des maladies cardiovasculaires dont le stade ultime est l'insuffisance cardiaque;
- Sa prise en charge repose essentiellement sur le support respiratoire, les vasodilatateurs, les diurétiques, les médicaments à effet inotrope positif en plus du contrôle du facteur de décompensation.

### Contribution de notre étude à la connaissance

- Le profil clinique de l'insuffisance cardiaque aiguë vue au niveau des services des urgences est différent de celui des services de cardiologie;
- L'utilisation de protocole standardisé de prise en charge thérapeutique dans l'insuffisance cardiaque aiguë basé essentiellement sur le contrôle de la défaillance respiratoire permet de mieux contrôler la morbi-mortalité de cette pathologie et de diminuer la durée d'hospitalisation et le coût;
- Le traitement initié rapidement depuis le service d'urgence permet d'améliorer le pronostic de cette pathologie.

## Conflits d’intérêts

Les auteurs ne déclarent aucun conflit d'intérêts.
